# Assessment of Animal-Based Pig Welfare Outcomes on Farm and at the Abattoir: A Case Study

**DOI:** 10.3389/fvets.2020.576942

**Published:** 2020-10-05

**Authors:** Dayane Lemos Teixeira, Laura C. Salazar, Daniel Enriquez-Hidalgo, Laura A. Boyle

**Affiliations:** ^1^Instituto de Ciencias Agroalimentarias, Animales y Ambientales (ICA3), Universidad de O'Higgins, San Fernando, Chile; ^2^Departamento de Ciencias Animales, Pontificia Universidad Católica de Chile, Santiago, Chile; ^3^Bristol Veterinary School, University of Bristol, Langford, United Kingdom; ^4^Sustainable Agriculture Sciences Department, Rothamsted Research, North Wyke, Okehampton, United Kingdom; ^5^Pig Development Department, Teagasc Animal and Grassland Research and Innovation Centre, Fermoy, Ireland

**Keywords:** pig, welfare, lesion, inspection, ante-mortem (AM)

## Abstract

The study assessed the prevalence of animal-based pig welfare outcomes on one Chilean farm and one abattoir. A total of 198 pens of slaughter pigs (9,049 pigs) were observed on farm and 54 batches (8,843 pigs) were observed at the abattoir. All assessments were conducted from outside the pen on farm and from outside the corridor where pigs were unloaded from the truck at the abattoir. Batch size and number of pigs with ear, tail and skin lesions, hernias, rectal prolapse, bursitis, and lameness were recorded. Data were analyzed using generalized linear mixed models. There was a large variation among pens on farm and among batches at the abattoir for all outcomes. Bursitis was the most prevalent outcome recorded in both locations, followed by ear lesions recorded on farm and by tail lesions recorded at the abattoir. Ear lesions' prevalence was higher on farm (*P* < 0.001), while tail lesions, hernia, and bursitis prevalence were higher at the abattoir (*P* < 0.001). Ear lesions' prevalence on farm was higher in female and mixed-sex groups than in male groups (P < 0.01), but male groups tended to have a higher tail lesions' prevalence (*P* < 0.1). The results show a difference in welfare outcomes, suggesting that assessment of outcomes on farm could complement ante-mortem inspections at the abattoir. However, as the same animals were not inspected in the two locations and there is the possibility of a seasonal influence on the results, the findings should be interpreted with caution and further research is required.

## Introduction

The primary function of ante- and post-mortem inspection is the protection of public health by ensuring food safety (1). As part of this process, the detection of illness or injuries during ante-mortem and lesions during the post-mortem inspection can lead to whole or partial condemnation of carcasses. There is considerable variation in the amount and quality of ante- and post-mortem data available internationally (2). However, it can play a valuable role in reducing financial losses (3, 4) and better informing herd health and welfare management plans (5). These data are routinely collected for disease surveillance (1), but they are also used in epidemiological studies to investigate risk factors (6), farm performance indicators (7), geographical or seasonal differences (8), and variation between herds (9). Furthermore, there is growing interest in the collection of information relating to welfare of animals at meat inspection (10, 11).

Nonetheless, death or euthanasia of severely affected pigs before slaughter could mean an underrepresentation of important data relevant to pig welfare collected at the abattoir. Hence, although meat inspection carried out at the abattoir offers an ideal opportunity for continuous and practical measurement of health and lesions, the prevalence of lesions detected is unlikely to be an exact representation of the extent of the problem on farm. Furthermore, some researchers have concerns about the probability of detecting anomalies during routine ante-mortem inspection at the abattoirs (12–14), arguing that time constraints, overcrowding, poor lighting, soiled hides, smell and noise pose challenges (14). On farm welfare assessments are commonly done daily and before loading [e.g., (15)] and could help overcome concerns related to ante-mortem inspection at the abattoirs. Importantly, on farm inspection can help with segregation of pigs that are at high-risk for gross abnormalities (13, 16). For example, suspect pigs have higher risk of suffering transport injuries than normal pigs (13); therefore, on farm pre-selection of suspect pigs could facilitate (rather than replace) abattoir-based ante-mortem inspection and reduced meat loss due to increased risk for injuries by separating pigs prior to transport into groups with and without visible lesions (17). Finally, sick and injured animals could be detected in advance, with the potential to prevent animals that are not fit for slaughter or for transport being sent to the abattoir (5). The aim of the current study was to assess animal-based pig welfare outcomes on farm and the abattoir.

## Materials and Methods

This was an observational study whereby pigs were managed according to routine practices on one Chilean commercial farm and in one commercial abattoir. The farm (May to October 2018) and the abattoir (October 2018 to May 2019) were visited over 8 and 34 days, respectively, and a welfare assessment of slaughter pigs was conducted. Animals inspected at the abattoir belonged to the same farm, but the same animals were not inspected in the two locations. Each visit (on farm and at the abattoir) lasted between 5 and 7 h.

A total of 198 pens of female and castrated male slaughter pigs were observed on farm accounting for a total of 9,049 pigs. The average group size was 45.7 (*SD* 4.85 pigs/pen; range 17–54); most of the pigs were kept on farm in single sex groups: 42.9% were female, 47% were male, and 10.1% were mixed-sex groups. Pens were selected on farm using proportionate stratified sampling to ensure that different environmental and pen characteristics were equally represented. Hospital pens on farm were excluded to ensure that pens were representative of the general population of the farm.

At the abattoir, 54 batches were observed accounting for a total of 8,843 pigs. The average batch size was 163.8 (SD 39.74 animals/batch; range 25–200). Pigs were mixed before being transported and arrived at the abattoir in mixed-sex groups. Transport system was the same for all batches. All animals observed were tail docked (5–10 cm) following normal management at Chilean farms. Batches evaluated were part of a larger experiment that aimed to investigate the association between ante- and post-mortem inspection welfare outcomes of slaughter pigs. Animals were transported and handled according to normal routine. The transports were conducted between the farm and the slaughterhouse 120 km away, accessed by motorways and secondary roads. The journey took approximately 2 h and 30 min.

Two observers (a veterinarian and a veterinary technician) were trained at the beginning of the study to ensure inter-observer reliability. All assessments were conducted from outside the pen on farm and from outside the corridor where pigs were unloaded from the truck at the abattoir. This was in accordance with how stock people and veterinary inspectors commonly inspected pigs at the two locations [adapted from (18)]. The batch size and the number of pigs with ear lesions, tail lesions, skin lesions, hernias, rectal prolapse, bursitis and lameness [adapted from (18)] were recorded (Table 1). Only severe cases of ear, tail and skin lesions and lameness were considered. Unfortunately, it was not possible to assess both sides of the pigs' body to detect the presence of certain animal-based welfare outcomes (e.g., skin lesions, bursitis), both on farm and at the abattoir assessments.

**Table 1:** Animal-based welfare outcomes recorded during welfare assessment on a commercial farm and a commercial abattoir [adapted from (18)].

**Outcome**	**Description**
Ear lesions	Bloody, swollen, and/or amputated ear
Tail lesions	Bloody, swollen, and/or amputated tail
Skin lesions	Presence of deep wound and/or hematoma
Hernia	Umbilical, scrotal, and/or inguinal hernia
Rectal prolapse	Internal tissue extrudes from the rectum
Bursitis	Presence of inflamed bursae (tangerine-sized or larger) on at least one limb(s)
Lameness	Very reluctant to walk, minimal weight-bearing on the affected limb or inability to move

*Only severe cases of ear, tail and skin lesions and lameness were considered*.

### Statistical Analysis

Animal-based welfare outcomes were recorded as the number of pigs affected per pen or batch and expressed as the percentage of pigs affected for each outcome. The median and interquartile range (IQR) of the prevalence of the different animal-based welfare outcomes were calculated for farm and abattoir assessments and ranked to identify the most prevalent outcomes within the two measurements. The number of pens where at least one animal was affected by each animal-based welfare outcome was identified to evaluate whether certain outcomes might be lowly prevalent but spread over the farm. Due to the very low prevalence of skin lesions and rectal prolapse they were not analyzed further. All statistical analyses were conducted using SAS 9.3 (SAS Institute, Inc., Cary, NC, USA). The effect of assessment location and animal sex on the prevalence of each separate animal-based welfare outcome were analyzed using generalized linear mixed models (Proc Glimmix). Assessment location (*n* = 252) and animal sex (*n* = 198; abattoir assessments excluded) were included in the models as fixed effects and gamma as the type of distribution. Least squares means (95% CI) are presented. Statistical effects and tendencies were reported when *P* < 0.05 and *P* < 0.10, respectively.

## Results

### Prevalence of Animal-Based Welfare Outcomes Assessed on Farm and at the Abattoir

The number and percentage (%) of pens on farm (*n* = 198) and batches at the abattoir (*n* = 54) with at least one pig affected by animal-based welfare outcomes is shown in Table 2. In general, a large variation was observed among pens on farm and among batches at the abattoir for all animal-based welfare outcomes. Bursitis was the most prevalent outcome recorded in both locations, followed by ear lesions recorded on farm and by tail lesions recorded at the abattoir.

**Table 2:** Number and percentage (%) of pens on farm (198 pens; 9,049 pigs) and batches at the abattoir (54 batches; 8,843 pigs).

	**On farm**				**At the abattoir**			
	**Pens**		**Prevalence**		**Batches**		**Prevalence**	
	***n***	**%**	**Median %**	**IQC %**	***n***	**%**	**Median %**	**IQC %**
Ear lesions	74	37.4	0.0	0.0–2.3	11	20.4	0.0	0.0–0.0
Tail lesions	31	15.7	0.0	0.0–0.0	40	74.1	2.8	0.0–9.44
Skin lesions	0	0.0	0.0	0.0–0.0	12	22.2	0.0	0.0–0.0
Hernia	25	12.6	0.0	0.0–0.0	22	40.7	0.0	0.0–0.6
Rectal prolapse	2	1.0	0.0	0.0–0.0	6	11.1	0.0	0.0–0.0
Bursitis	170	85.9	6.5	4.1–9.3	54	100.0	11.7	8.3–13.9
Lameness	24	12.1	0.0	0.0–0.0	6	11.1	0.0	0.0–0.0

*With at least one pig affected by animal-based welfare outcomes, including the median prevalence and interquartile range (IQR) of pigs affected per pen and per batch (%) of each animal-based welfare outcome observed on the two locations*.

### Effect of Assessment Location and Animal Sex

The effect of assessment location and animal sex on the prevalence of animal-based welfare outcomes assessed on farm and at the abattoir is presented in Table 3. Detected prevalence of ear lesions was higher on farm compared to the abattoir assessments (*P* < 0.001). In contrast, detected prevalence of tail lesions, hernia and bursitis were higher at the abattoir than on farm (*P* < 0.001). Within on farm assessments, the prevalence of ear lesions was higher in female and mixed-sex groups than in male groups (*P* < 0.01), but male groups tended to have a higher prevalence of tail lesions than the other groups (*P* < 0.1).

**Table 3:** Effect of assessment location and animal sex on the prevalence of animal-based welfare outcomes in slaughter pigs assessed on farm (198 pens; 9,049 pigs) and at the abattoir (54 batches; 8,843 pigs).

	**Location**			**Sex**^****a****^			
	**Farm**	**Abattoir**	***P*-value**	**Female**	**Male**	**Mixed**	***P*-value**
Ear lesions	2.1 (1.92–2.31)	−0.3 (−0.77 to 0.23)	***	2.3 (2.05–2.53)^a^	1.3 (0.94–1.70)^b^	2.2 (1.73–2.71)^a^	**
Tail lesions	1.5 (1.11–1.84)	2.5 (2.14–2.78)	***	1.9 (1.38–2.46)	1.3 (0.98–1.70)	0.9 (0.24–1.60)	+
Hernia	0.9 (0.74–1.17)	0.2 (−0.03 to 0.42)	***	1.0 (0.80–1.21)	0.9 (0.62–1.13)	0.9 (0.38–1.46)	ns
Bursitis	2.0 (1.96–2.11)	2.4 (2.31–2.59)	***	2.1 (1.96–2.21)	2.0 (1.85–2.09)	2.1 (1.84–2.37)	ns
Lameness	1.1 (0.88–1.38)	1.1 (0.65–1.64)	ns	1.3 (0.94–1.59)	0.8 (0.36–1.19)	1.3 (0.62–1.98)	ns

*Least squares means (95% CI) of models with gamma distribution are presented*.

a Abattoir assessments were excluded as sex of pigs were not assessed in this location; ns, not significant; +, Tendency (P < 0.1);

** P < 0.01;

*** *P < 0.001*.

## Discussion

The current case study provides results of animal-based welfare outcomes assessed in slaughter pigs on farm and at the abattoir. In general, the detected prevalence of animal-based welfare outcomes assessed in both locations was very low, which is positive for pig welfare. However, considering that only one commercial farm supplying one abattoir participated in the study, the findings should be interpreted with caution and does not represent a general or national figure. Also, it is important to highlight that the same animals were not inspected in the two locations, which was a major limitation in the methodology.

Our findings show a wide variation in the prevalence of animal-based welfare outcome measures among pens on farm and among batches at the abattoir, which could suggest that different risk factors on farm and during transit might play a role and may contribute to the variation observed. Previous studies reported large variation in the prevalence of welfare outcomes between different ages/weight groups of pigs (18–20) and between countries (10, 21). It is important to note that all animals from our study were slaughter pigs with similar age and body weight.

In accordance with other studies carried out in Spain and Ireland (18, 22), bursitis was one of the most prevalent outcomes observed, even that these authors only recorded severe cases. The prevalence of bursitis was higher at the abattoir than on farm assessments, probably because the animal legs were more visible to the observer's eye while the animals were walking down from the truck rather than while grouped together in the pens on farm. Prevalence of ear and tail lesions was lower than reported by Petersen et al. (19) in Denmark and van Staaveren et al. (18) in Ireland in finishing pigs; however, similar trends for tail lesions were found by Temple et al. (22) in growing pigs in Spain. The prevalence of ear lesions on farm was higher than tail lesions, which is in agreement with van Staaveren et al. (18); while van de Meer et al. (23) found that ear biting was scored more frequently than tail biting behavior. The higher prevalence of tail lesions reported at the abattoir than in farm assessments seems to be due to the fact that hospital pens were not included in the on farm assessments but pigs from hospital pens could have been mixed with healthy pigs prior to transportation from farm to the abattoir.

Furthermore, different challenges were faced during assessments on farm and at the abattoir. On farm animals were sometimes huddling or lying down and at the abattoir the speed of unloading was often very fast. These must be considered when comparing the findings of welfare assessments on farm with assessments conducted at the abattoir. These observations are in accordance with other studies that expressed concerns regarding the time constraints and overcrowding during ante-mortem inspection at the abattoirs (12–14).

Moreover, almost 50% of the pens assessed on farm were all male groups, in which pigs presented lower prevalence of ear lesions and tended to present a higher prevalence of tail lesions than pigs in female and mixed-sex groups. In accordance with previous studies (3, 4, 24), males are more frequently affected by tail lesions than females and this trend becomes exaggerated with increasing tail lesion severity. Females tend to perform more tail-in-mouth behavior than male pigs (25), however, to our knowledge, the sex effect on ear lesions was not reported previously. The higher prevalence of ear lesions in groups of female pigs could suggest that they have a higher propensity to bite in general and to direct biting behavior toward the opposite sex (25), which is supported by our findings where the high prevalence of ear lesions was also found in mixed-sex pens. On the other hand, the etiology of ear lesions/necrosis is not elucidated yet and the sex effect could also be associated to immune susceptibility of female pigs to subsequent bacterial infection on the damaged tissue.

The period of the year and, consequently, the season of assessments on farm and at the abattoir differed greatly in the current study, which was also a limitation in the methodology. The prevalence of lesions and diseases is known to vary with season (8, 26). There is a higher prevalence of ear biting (27) in the winter months than during summer, which could explain our findings of higher prevalence of ear lesion on farm than at the abattoir assessment. Seasonal influence on tail lesion is also reported (27, 28), suggesting that heat- or cold-stressed pigs are more prone to perform tail biting (26).

The results from our study suggest that farm based assessments could augment the information collected at the abattoir ante-mortem inspection but further research following the same group of animals longitudinally from farm to the abattoir is required to confirm such assumption. Our findings also support that it is possible to identify animals with health and welfare outcomes on farm and to transport them from farm to abattoir in a separate group (13), allowing meat inspection procedures to be made more efficient (16) and to decrease the risk of microbial cross-contamination (5). Additionally, such animal-based welfare outcomes can be incorporated with food chain information (FCI) (29, 30), enabling evidence-based risk categorization of pigs before slaughter (31).

## Conclusion

The results from our study show a difference in animal-based welfare outcomes, suggesting that assessment of animal-based pig welfare outcomes on farm could complement ante-mortem inspections at the abattoir. However, due to the use of a convenience sample, the same animals were not inspected in the two locations and the possibility of seasonality influence on the results, the findings should be interpreted with caution and further research following longitudinally the same group of animals from farm to abattoir is requited to confirm such assumption.

## Data Availability Statement

The raw data supporting the conclusions of this article will be made available by the authors, without undue reservation.

## Ethics Statement

This study was part of a research project approved by the Scientific Ethics Committee for Animals and Environmental Care of the Pontificia Universidad Católica de Chile (Protocol No. 170529006). Written informed consent was obtained from the owners for the participation of their animals in this study.

## Author Contributions

DT, LS, LB, and DE-H contributed to the concept of the work. DT and LS initiated and designed the study and performed the experiment. DT and DE-H performed statistical analysis. DT, DE-H, and LB interpreted data. DT wrote the manuscript. DE-H, LS and LB, contributed to the manuscript. All authors contributed to the article and approved the submitted version.

## Conflict of Interest

The authors declare that the research was conducted in the absence of any commercial or financial relationships that could be construed as a potential conflict of interest.
